# Piezoelectric Particulate Composite for Energy Harvesting from Mechanical Vibration

**DOI:** 10.3390/ma13214925

**Published:** 2020-11-02

**Authors:** Dariusz Grzybek, Dariusz Kata, Wojciech Sikora, Bogdan Sapiński, Piotr Micek, Hanna Pamuła, Jan Huebner, Paweł Rutkowski

**Affiliations:** 1 Faculty of Mechanical Engineering and Robotics, AGH University of Science and Technology, Al. Mickiewicza 30, 30-059 Krakow, Poland; wosikora@agh.edu.pl (W.S.); deep@agh.edu.pl (B.S.); micek_pt@agh.edu.pl (P.M.); pamulah@agh.edu.pl (H.P.); 2 Faculty of Materials Science and Ceramics, AGH University of Science and Technology, Al. Mickiewicza 30, 30-059 Krakow, Poland; kata@agh.edu.pl (D.K.); huebnerj@agh.edu.pl (J.H.); pawelr@agh.edu.pl (P.R.)

**Keywords:** piezoelectric energy harvesting, piezoelectric particulate composite, PZT particulate-epoxy composite, building vibration, bending mode

## Abstract

Energy harvesting from mechanical vibration of buildings is usually realized by the use of devices, in which the main element is a prismatic beam with a rectangular cross-section. The beam has been the subject of scientific research; it is usually constructed with a carrying substrate that does not have piezoelectric characteristics and from piezoelectric material. In contrast, this investigation sought to create a beam structure with a piezoelectric composite only. The entire beam structure was made of a prototype piezoelectric particulate composite. Based on courses of voltage obtained in laboratory experiments and known geometry of the specimens, a series of finite element method (FEM) simulations was performed, aiming to estimate the piezoelectric coefficient *d*_31_ value at which the mentioned voltage could be achieved. In each specimen, sedimentation caused the formation of two distinct layers: top and bottom. The experiments revealed that the presented prototype piezoelectric particulate composite converts mechanical stress to electric energy in bending mode, which is used in energy harvesting from mechanical vibration. It is self-supporting and thus a carrying substrate is not required in the harvester structure.

## 1. Introduction

Piezoelectric energy harvesting is realized by the use of specific devices, which are commonly called energy harvesters. The structure of the energy harvester depends on the source of mechanical energy, which may be building vibrations [1], motion of machines [2], ocean waves, wind energy, acoustic energy, or biomechanical reactions [3]. Piezoelectric energy harvesting from building vibration is usually realized by the use of a prismatic beam with a rectangular cross-section, which works in bending mode [4]. The prismatic beam is made from piezoelectric material wherein electrical energy is created as a result of deformations caused by reactions of vibrating mechanical structures. The beams are constructed from two materials: (1) a carrying substrate, which does not possess piezoelectric features; and (2) piezoelectric material, which is a composite made from piezoelectric ceramics and a polymer matrix, e.g., macro fiber composite [5,6], piezoelectric polymer, e.g., polyvinylidene fluoride [4,7]. The composite has lower energy converting efficiency than the sinters of piezoelectric ceramic, however, it is more resistant than monolithic ceramic to destruction owing to significant deformations, caused by strong tension [8]. Steel [1], aluminum [4,5], and brass [9] are the typical carrying materials used in beam structures. Such typical carrying is nowadays replaced with laminate material [10], carbon fibers [6,11], and other substrates. The beam structure is made by gluing of the above-mentioned piezoelectric materials and a carrier. Glue connections in the beam, however, tend to degrade when influenced by changeable tensions and external factors, and this process is random in nature, whose run cannot be fully predicted.

In contrast to these approaches, the present investigation created a beam structure containing only piezoelectric composites. Particulate composite (0–3) was selected for the research based on literature review. 0–3 particulate composites consist of randomly dispersed piezoelectric grains in a polymer matrix. Piezoelectric properties depend on a volume fraction of piezoelectric grains. Values of piezoelectric coefficient d_33_ showed a linear increase due to rise in piezoelectric volume fraction [12]. However, piezoelectric properties of e.g., PZT/PVC particulate composites show a decreasing trend when the content of lead zirconate titanate (PZT) exceeds 50% [13]. The dielectric constant of the piezoelectric particles also has a significant role in determining the piezoelectric properties of manufactured composites. Composites with a lower dielectric constant of PZT particles showed better piezoelectric properties, compared to composites with a higher constant [14]. The values of piezoelectric coefficients can be increased by the dielectrophoresis process, which enables the creation of a quasi 1–3 structure from the 0–3 composite [15,16]. The dielectrophoresis process can, therefore, be widely employed to create an intermediate state between a 0–3 and 1–3 connectivity pattern by manipulating particles in the composite through application of an alternating current (AC) electric field [17]. Improvement in piezoelectric properties of the quasi 1–3 structure can be achieved by a combination of alignment of the ceramic particles and poling process simultaneously while the polymer matrix is still in a liquid state [18]. Efficiency of the piezoelectric coefficient of particulate composites are mostly determined in compression mode [12,13,14,15,16,18] or less often in shear mode [19]. The determination of the piezoelectric coefficient of self-supporting particulate composites in bending mode has not been explored yet.

This paper describes the investigation into energy harvesting from mechanical vibrations with the use of beams, the whole structure of which is made of self-supporting 0–3 piezoelectric particulate composite. The piezoelectric properties of the composite did not improve with the use of the dielectrophoresis process. In contrast to the above-mentioned approaches, this particulate composite was found to work in the bending mode. 

## 2. Materials and Methods 

### 2.1. Manufacturing of Piezoelectric Material

**Figure 1:**
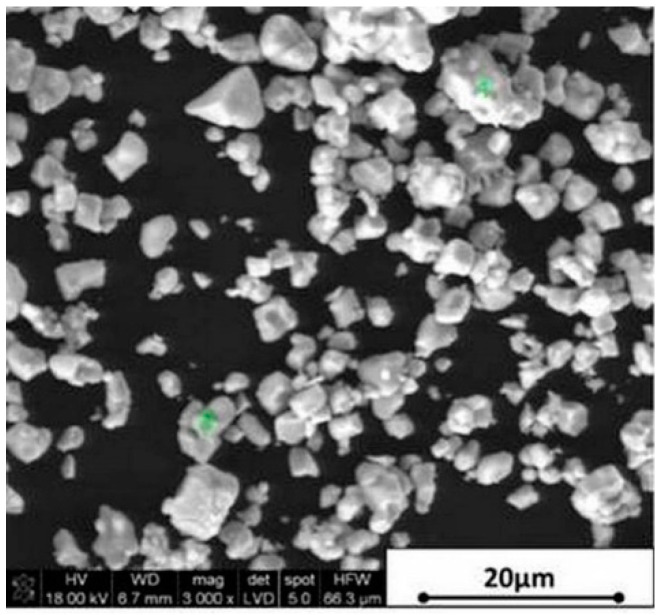
Morphology of PZT commercial powder used for the production of particulate piezoelectric composite.

The subject of laboratory and numerical research was a prismatic beam, manufactured from piezoelectric particulate composite. Two commercial components were used to prepare the composite: (1) PZT powder, the grain dimensions of which did not exceed 5.0 μm, and (2) a chemically initiated methacrylate resin (Duracryl Plus, manufactured by SpofaDental, Jičín, Czech Republic). Duracryl Plus consisted of two components: powder and liquid monomer. Morphology of the PZT powder is presented in Figure 1.

**Figure 2:**
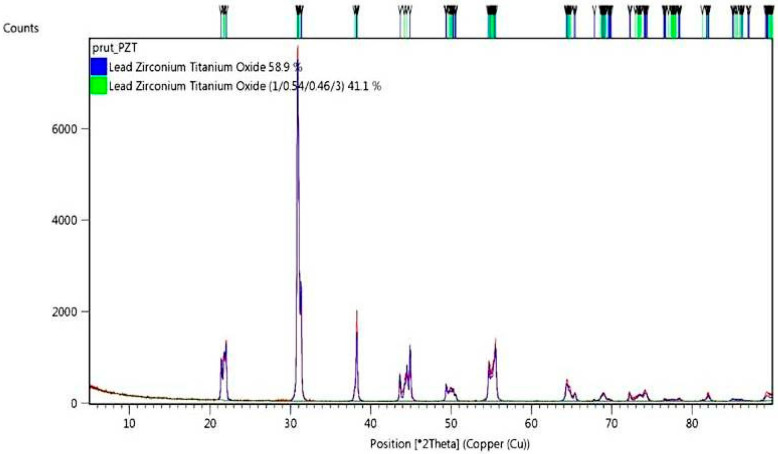
X-ray diffraction (XRD) pattern of the as-received PZT commercial powder.

Phase composition of PZT is presented in Figure 2. The XRD diffraction pattern was carefully examined and matched to the peak position of the commercially delivered PZT powder. The analysis was done to check for quality control of the as-received powder. As per the supplier, the powder was certified to have impurity content less than 0.5%. The investigation revealed minor peaks related to the impurities; however, there was no point in analyzing them due to the XRD detection level. The diffractogram peaks were analyzed based on the available database of diffractograms of chemical compounds.

**Figure 3:**
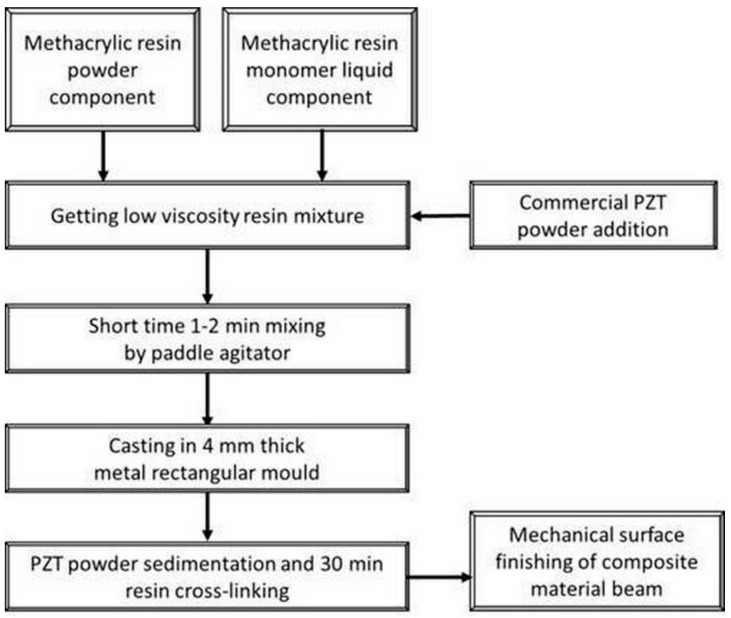
Schematic flow chart of the preparation method of the low cost particulate nanolaminate polymer-ceramic piezoelectric beam.

The manufacturing process included a sedimentation process, which is undesired for rheological stable slurries. This phenomenon, however, turned out to be an ally. Because of the bi-modal powder size distribution (as shown in Figure 1) and agglomeration effect, larger PZT particles and agglomerates quickly sank to the bottom of the liquid resin. Due to viscosity and rate of cross-linking of the resin, very fine particles remained in the upper layer of the produced beam, creating randomly distributed conductive paths. The resin curing time was 33 min in total, following which PZT particles were found to be embedded in the cured resin. In summary, by optimizing the physical sedimentation process, appropriate selection of the liquid component of the resin, and the use of high specific density PZT particles made it possible to obtain a piezoelectric beam composite. A schematic flow chart of the preparation of the low-cost particulate nanolaminate polymer-ceramic piezoelectric beam is shown in Figure 3.

**Figure 4:**
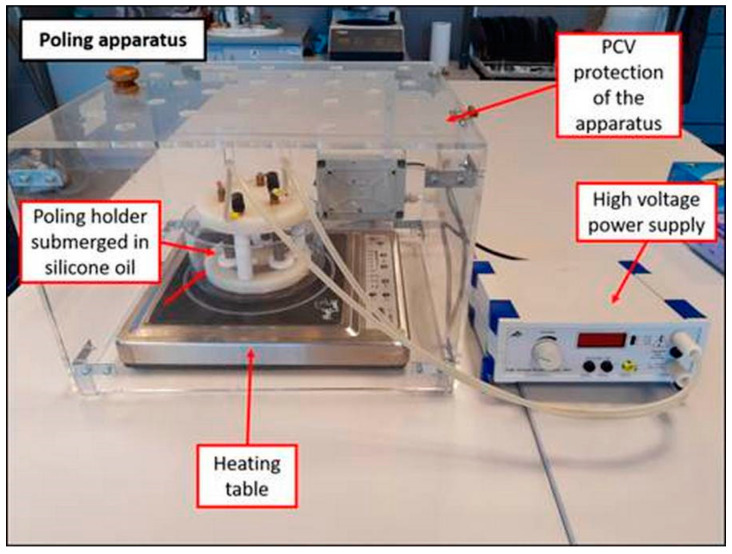
Prototype apparatus used for polarization of the manufactured piezoelectric beam.

The manufacturing process of the prismatic composite beam for energy harvesting had the following stages:

1. Mixing the PZT grains and Duracryl Plus powder in a rotational dissolver, manufactured by Pendraulik-Teja, for 30 min. The volume fraction of PZT grains in the mixture was 50% and 40%. Liquid monomer was added during the mixing process.
2. Creating the prismatic beam in a special prototype metal mold. The general dimensions of the formed beam were permanent: length 110 mm, width 28 mm. The value of thickness depended on the volume fraction of the PZT powder.
3. Polishing the composite beam surfaces by the Rotopol-22 Struers device.
4. Covering the composite beam surface with two copper electrodes.
5. Poling the composite beam in a special prototype polarization stand, given in Figure 4.

The maximum applied voltage during poling was 4 kV. For the safety of the operator of the device, the system is electrically isolated through a polycarbonate chamber. Unintentional opening of the chamber causes the electrical contractor to close, therefore disconnecting the power supply. During the polarization process, the device is placed under the fume extractor. The maximum possible temperature and duration of the process were optimized experimentally in order to prevent deformation of the obtained material.

**Figure 5:**
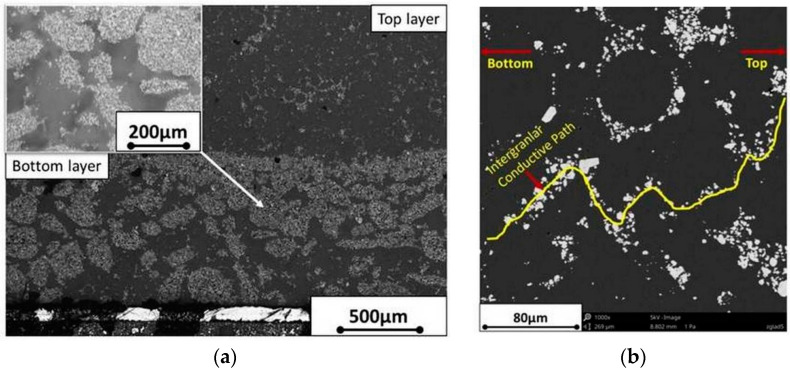
Cross-sections of the manufactured composite beam: (**a**) cross-section of both top (polymer) and bottom (piezoelectric) layer observed by optical microscope; (**b**) SEM cross-section observations of only the top (polymer) layer with PZT fine particles distributed in the matrix building conductive paths.

On the basis of microscope analysis, it was noted that the manufactured composite beam had a layered structure created by PZT particles’ sedimentation. Two main layers were visible (Figure 5a). The top layer was the resin with well-dispersed low concentration fine PZT particles. These particles created piezoelectric paths between the bottom layer and copper on the top layer (Figure 5b).

For better understanding, the top layer is referred to as the “top (polymer) layer” in the following chapters. The bottom layer contained large sedimented particles from the ceramic piezoelectric phase in the resin. The bottom layer is referred to as the “bottom (piezoelectric) layer” in the next chapters. After the casting and cross-linking processes (Figure 3), average thickness of the bottom layer was 989 ± 23 µm and the sample thickness was 3479 ± 70 µm. The sample’s surface was ground and smoothened to reach sample thickness of 2.7 mm; it was 1.85 mm in the polishing stage. These dimensions were measured in six cross-section places on the 11 cm sample in 1 cm steps. 

### 2.2. Methods Used in Laboratory Research

**Figure 6:**
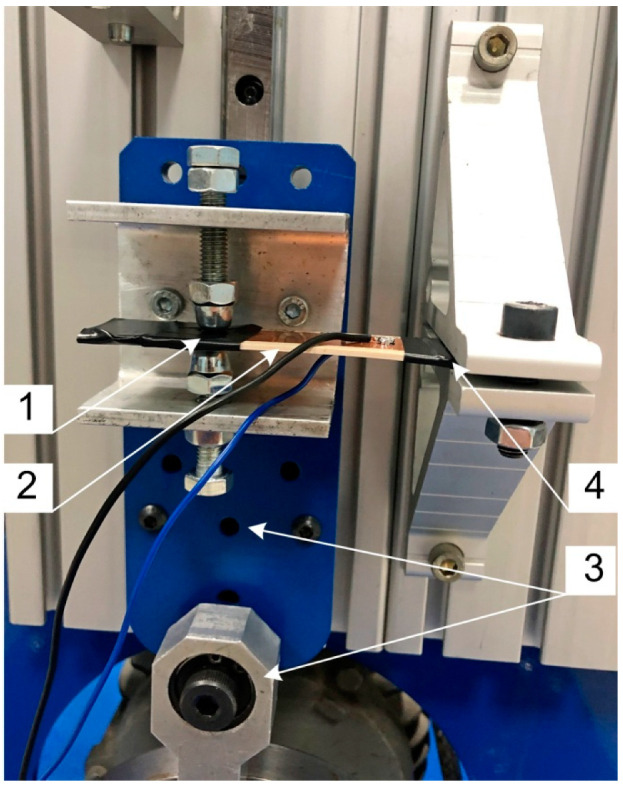
Laboratory setup used in laboratory research: 1—moving end of the composite beam, 2—particulate composite beam, 3—vibration generation system, 4—stationary end of the composite beam.

A specially designed experimental setup for beam bending (Figure 6) was used to assess the signal generated by the manufactured composite beam. 

The moving end (1) of the manufactured composite beam (2) was fixed to a vibration generation system (3). The stationary end (4) was fixed to a immobile laboratory stand. The vibration generation system enabled sinusoidal displacement of the moving end of the composite beam. The experiments were conducted for different values of vibration amplitude, frequency, and load resistance. The peak-to-peak amplitude of the sinusoidal displacement of the moving end was: 6 mm, 10 mm, and 12 mm. The laboratory experiments were conducted for three vibration frequencies: 10 Hz, 15 Hz, and 20 Hz and for six values of load resistance: 0.97 MΩ, 1.84 MΩ, 2.63 MΩ, 4.03 MΩ, 5.23 MΩ, and 6.24 MΩ. The load resistance was connected in parallel to the manufactured beam. The data acquisition board DAQ 2000 was used to digitize the signal and send it to the personal computer. The voltage signal analysis was performed in DasyLab software (5.02 version, Measurement Computing Corporation, 10 Commerce Way, Norton, MA, USA). To get rid of the electrical noise (50 Hz), we used a 5th order low-pass Butterworth filter with 50 Hz cut-off frequency.

### 2.3. Methods Used in Numerical Research

Numerical simulations using the finite element method were performed in an ANSYS environment. The aim was to estimate the piezoelectric coefficient of the investigated composite beam by performing inverse calibration. Based on values of the voltage obtained in the laboratory experiments and known geometry of the specimens, a series of FEM simulations were performed, which aimed at estimating piezoelectric coefficient *d*_31_ value for which the voltage could be achieved.

**Figure 7:**
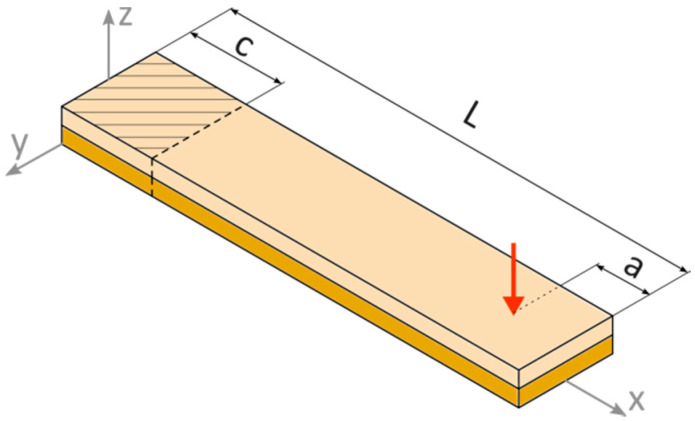
Configuration of the particulate composite beam in FEM simulations: L—length of beam, a—distance between the moving end of the beam and application point of the external kinematic load, c—clamped part of the beam.

General configuration of the model is presented in Figure 7, corresponding to conditions of the laboratory experiments. The part of the specimen that was clamped in the FEM model, here described as length ‘c’, was fixed to its top and bottom surfaces. An external kinematic load was applied, similar to the laboratory experiments, at a point receded by length ‘a’ from the beam moving end. General dimensions of the beam were the same as the laboratory experimental specimens: L = 110 mm, c = 25 mm, and a = 23 mm, while total thickness varied depending on the specimen PZT content. 

**Figure 8:**
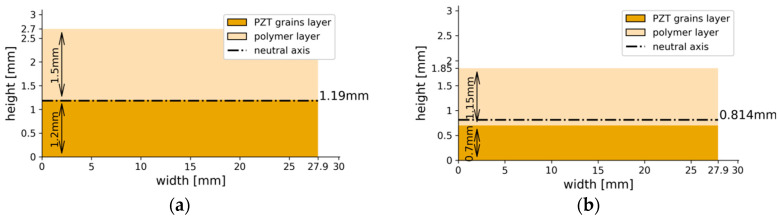
Schematic representing the cross-sections of the particulate composite beam: (**a**) beam containing initial 50 vol. % PZT; (**b**) beam containing initial 40 vol.% PZT.

Cross-sections of each of the tested composite specimens are presented in Figure 8.

## 3. Results

### 3.1. Results of the Laboratory Research

**Figure 9:**
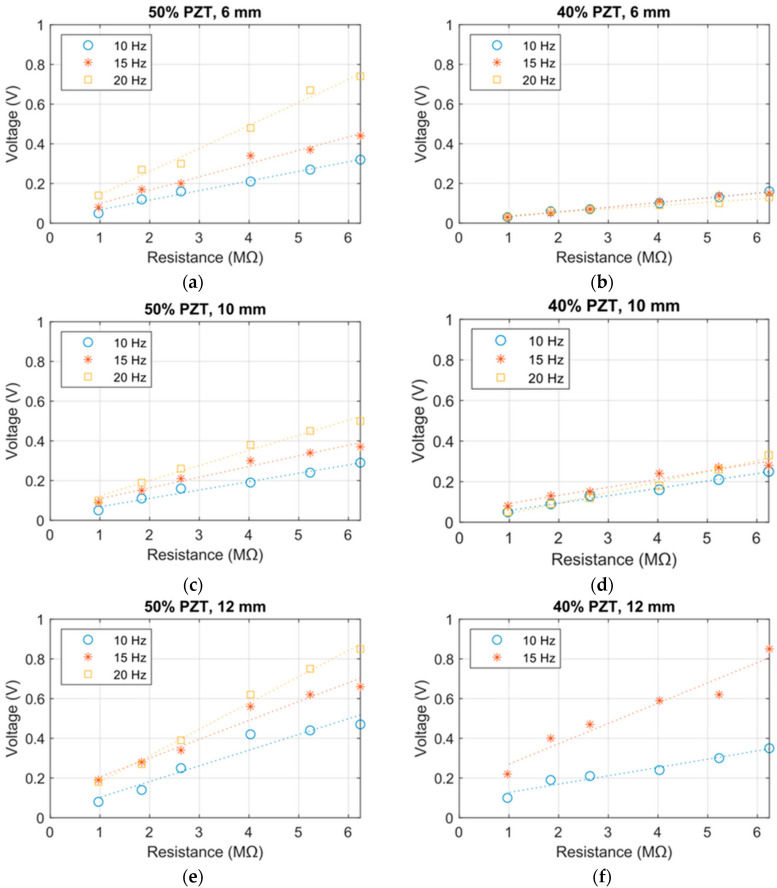
Courses of voltage generated by the manufactured beam: (**a**) initial volume share of PZT: 50% and vibration amplitude: 6 mm; (**b**) initial volume share of PZT: 40% and vibration amplitude: 6 mm; (**c**) initial volume share of PZT: 50% and vibration amplitude: 10 mm; (**d**) initial volume share of PZT: 40% and vibration amplitude: 10 mm; (**e**) initial volume share of PZT: 50% and vibration amplitude: 12 mm; (**f**) initial volume share of PZT: 40% and vibration amplitude: 12 mm (missing the 20 Hz measurements plot, as the specimen broke during the experiment).

Courses of voltage generated by the particulate composite beam were obtained by the laboratory experiments. The composite beam (containing initial 50 vol. % PZT or 40 vol. % PZT) was tested for three values of vibration frequencies: 10 Hz, 15 Hz, and 20 Hz and six values of load resistance: 0.97 MΩ, 1.84 MΩ, 2.63 MΩ, 4.03 MΩ, 5.23 MΩ, and 6.24 MΩ in each experiment. Output voltage was measured as the peak-to-peak amplitude of the filtered voltage signal. The obtained courses of voltage are presented in Figure 9.

The results of the laboratory experiments confirm the hypothesis on generation of electric energy by the beam, whose entire structure is made of a PZT particulate-epoxy composite. Values of the generated voltage are dependent on the volume fraction of the PZT grains and on the frequency of bending of the composite beam. These results show that the composite containing an initial 50 vol.% of PZT generated the highest values of voltage. 

### 3.2. Results of the Numerical Research

The modelling approach adopted in the FEM simulations was similar for a layered composite. These two earlier mentioned layers were distinguished by attributing different material properties to each of them. It was assumed that the bottom (piezoelectric) layer is the active one and responsible for piezoelectric conversion during beam loading. As the specimen functions in bending mode, only the *d*_31_ piezoelectric coefficient is taken into account; its value was estimated during the simulations. The effects of other coefficients d_33_ and d_15_ are negligible and assumed to be zero. This layer is polarized in the z direction, same as that of the experimental specimen. The top (polymer) layer made mostly from polymer is treated as the standard material without piezoelectric properties. It acts as an insulator, which in part inhibits gathering of charge on the top electrode. Other material parameters used in the model are listed in Table 1. To estimate the elasticity modules E of both the main layers, the composite specimen had to be separated. Through grinding, the top (polymer) layer (visible in Figure 5b) was removed, leaving only the bottom (piezoelectric) layer with visible PZT grains. This sample then underwent a static bending test based on which the E modulus was estimated. A similar procedure was realized for the top (polymer) layer; however, in this case, a completely new sample from pure PMMA was prepared.

The sample did not contain fine ceramic particles, which was found in the top (polymer) layer of the composite (Figure 5b); however because of their size and amount, they were not expected to have a significant influence on the layer’s mechanical properties. Poisson ratios were approximated based on typical values for the PZT ceramics and PMMA polymer found in generally available sources [20]. The calculated first natural frequency in bending mode for the specimen constrained (as shown in Figure 7) is 51.1 Hz for the composite containing an initial 50% PZT and 38.3 Hz for 40% PZT.

The dielectric constant ε_r_ of the PMMA layer was assumed as being typical for this type of polymer [21]. Estimating the relative permittivity of the bottom (piezoelectric) layer is more complicated because it is a composite material. It consists of PZT grains within the polymer matrix, which sedimented during its curing. Even though the dielectric constants for both PZT and PMMA are known, estimating its effective value for the whole layer is not straightforward. There are several theoretical models [21,22,23] found in the literature that aim to estimate the effective dielectric constant of the 0–3 polymer-ceramic composites. The cited works, including [24], point out the models’ general inaccuracies; therefore, their predictions should be interpreted as approximations. Among the described models, the Maxwell-Garnett model was chosen to calculate effective permittivity as it relies on currently known parameters like volume fraction and dielectric constants of the layer’s two components. According to the Maxwell-Garnett model, effective permittivity *ε_eff_* can be calculated by the following equation:

(1) $$\varepsilon_{eff}=\varepsilon_{m}\;\frac{2\varepsilon_{m}+\varepsilon_{i}+2v_{i}\left(\varepsilon_{i}-\varepsilon_{m}\right)}{2\varepsilon_{m}+\varepsilon_{i}-v_{i}\left(\varepsilon_{i}-\varepsilon_{m}\right)}$$

where:

*ε_m_*—dielectric constant of polymer (*ε_m_* = 3)
*ε_i_*—dielectric constant of PZT (*ε_i_* = 1700)
*v_i_*—volume fraction of PZT

**Figure 10:**
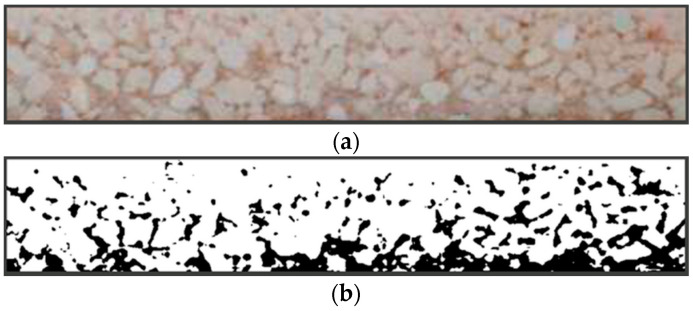
A cross-section of the composite sample narrowed on the bottom (piezoelectric) layer with densely packed PZT grains: (**a**) original image; (**b**) binary image.

During composite preparations, while the polymer was still in a liquid state, majority of the PZT particles sedimented at the bottom of the sample mold. Even though the components’ initial volume fractions are known (50% and 40% of PZT), due to the fact that ceramic powder settled on the bottom, final ratios of this bottom layer are different. An image of the cross-section with the isolated layer containing tightly packed PZT grains within the polymer matrix is shown in Figure 10a. To assess the ratio between the polymer and the PZT, a graphic analysis of the sections was performed. Using the color threshold method, a binary image (Figure 10b) of the section was obtained, from which the required ratios were estimated.

For the given samples, the mean PZT ratio in the bottom (piezoelectric) layers of the composites was assessed as *v_i_* = 74.3%. Therefore, the effective dielectric constant for the bottom (piezoelectric) layer was calculated as *ε_eff_* = 28.5.

The procedure to estimate the *d*_31_ parameter was as follows: for each of the experimental test runs characterized by composite sample type, loading conditions, measuring resistance, and obtained voltage amplitude, a twin FEM simulation was prepared. However, as *d*_31_ was unknown, its value was determined by a basic optimization algorithm [25]. The objective of the procedure was to find the difference between the experimental voltage amplitude value, and this was done by FEM simulation; it had to be minimized. As it was a one-dimensional problem, the final results for the obtained test run was obtained after a few simulation iterations.

Thus, a set of $d_{31}^{p}$ values describing properties of the bottom (piezoelectric) layer was obtained, which was then recalculated to the value corresponding to the entire composite section, according to the following equation [26]:

(2) $$d_{31}=\frac{1}{E_{eff}}\cdot \frac{t_{p}}{t}\cdot d_{31}^{p}\cdot E^{p}$$

where:

$E_{eff}$—effective Young modulus for the composite
$t_{p}$—thickness of the piezoelectric layer
t—total thickness of the composite
$d_{31}^{p}$—piezoelectric coefficient of the piezoelectric layer
$E^{p}$—Young modulus of the piezoelectric layer ($E^{p}=2.81\;\mathrm{GPa}$)

**Figure 11:**
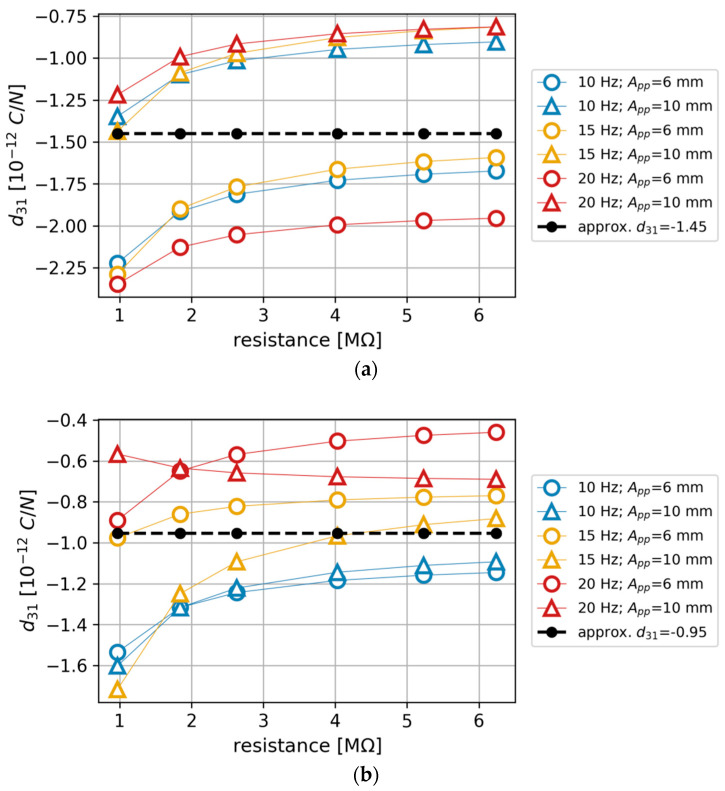
Values of the $d_{31}$ piezoelectric coefficient estimated from the FEM simulations (A_pp_ is a peak-to-peak excitation amplitude): (**a**) for the composite beam containing an initial 50% PZT; (**b**) for the composite beam containing an initial 40% PZT.

The results were divided into two parts as per the two variants of PZT content in the composite sample and presented in a graphical form in Figure 11.

Mean values of piezoelectric coefficient for each case were as follows:

- Beam containing an initial 50 vol. % PZT: *d*_31_ = −1.43 pC/N
- Beam containing an initial 40 vol. % PZT: *d*_31_ =−0.945 pC/N

If a given sample perfectly complies with the linear piezoelectricity model, the obtained *d*_31_ values should be identical. However, as can be seen in Figure 11a,b, the results show some deviations. The observed disparities are related to excitation amplitude and resistance value. Taking into account that the method (inverse FEM calibration) used to estimate *d*_31_ was an indirect one and the sample was in a bending mode (in which stresses are not uniform within the sample), some deviations are to be expected. Therefore, because in an ideal theoretical scenario, one constant value should be obtained for a sample, in the end, a mean value from all the results was calculated to approximate the *d*_31_ value for the given composite variant.

## 4. Discussion

The results of the laboratory research confirm the hypothesis of effective application of a beam made entirely of PZT particulate-epoxy composite for energy harvesting from mechanical vibrations. The experimental results for the beam containing an initial 50% PZT have shown that the higher the frequency of the vibration, the greater the voltage values were, up to 0.87 V for 20 Hz and 12 mm vibration amplitude. The general trend found in the results, considering the amplitude change, was as expected—increasing voltage with rise in amplitude. The beam containing an initial 40% PZT behaved in a less-consistent way than the beam containing an initial 50% PZT, generating similar outputs for three tested frequencies at lower amplitudes, but expectable values for the 12-mm amplitude. The inconsistencies between the specimens and the individual experiments may be the result of inhomogeneity of the samples or e.g., the mounting and clamping system in the laboratory setup.

Two types of composite beam specimens were modeled in this research work, which differed in initial PZT volumetric content: 50 vol. % and 40 vol. %. In each specimen, sedimentation of coarse fraction was observed, which caused the formation of two distinct layers. A section of two-composite type was recreated using FEM, and is presented in Figure 6. All the specimens functioned in the bending mode; thus, an important parameter of the cross-sections is the position of the neutral axis. As was discussed in [27], two factors influence its location: elasticity modulus and thickness of each layer. From the analysis of the sections in Figure 8, it can be expected that the composite containing an initial 50% PZT should generate a higher voltage than that containing initial 40% PZT because of two reasons: the PZT active layer is thicker and the neutral axis is located further from the active layer outer’s face in the first composite variant. This is confirmed by the comparison of voltage values: Figure 9a,b, and Figure 9c,d. With the bending, the distance from the neutral axis affects the strain, and the same stress is seen within the material. It is important to avoid cross-sections, where the neutral axis is located within the bottom (piezoelectric) layer, which is partially in tension and compression at the same time during bending. This results in the appearance of opposing electric fields and reduces the total piezoelectric effect, which should be avoided. On the other hand, the position of the neutral axis in the top (polymer) layer is also unfavorable, because a tensioned or compressed part of the beam will contain both the piezoelectric and polymer layer. The polymer layer does not generate electric energy. Hence, it is to be noted that the best position for the neutral axis is between the top and bottom layers, because a tensioned or compressed part of the beam will contain only a piezoelectric layer. Thus, the thickness of the top layer should be selected such that the neutral axis is between the top and bottom layers.

Taking into account the fact that polymer in the top layer does not generate electric energy, piezoelectric energy harvesting is only possible when piezoelectric paths that are created from ceramic grains exist in the top layer. Piezoelectric paths connect the bottom layer, which generates electric energy, with copper electrode on the surface of the top layer. It can be assumed that an increase in piezoelectric paths should cause an increase in electric energy, which is generated by the composite beam.

The investigated composite seems to be the 0–3 type with granular particles suspended in a polymer matrix. However, an interesting approach was taken while preparing the investigated material, where in the initially blended mixture, during the polymer curing phase, coarse ceramic particles were allowed to slowly sediment on the bottom of the molds. This led to obtaining a non-typical structure, which can be associated with both 0–3 (particles in matrix) and 2–2 (laminate) composites. For an energy harvesting purpose, especially when the bending mode is utilized, such a structure is highly promising, as the obtained material can be used directly, without the necessity of an additional carrying layer. Currently, the piezoelectric performance of the presented material shows room for improvement, which will be the aim of further research.

## 5. Conclusions

On the basis of laboratory and numerical experiments, it was found that the 0–3 piezoelectric particulate composite harvests electric energy in a bending mode. The bending mode is usually used in energy harvesting from mechanical vibrations of buildings. On the basis of the experiments, it was found that:

- A piezoelectric particulate composite, which contains two layers, harvests electric energy from mechanical vibration in a bending mode. The first layer should contain large particles of ceramic piezoelectric phase in the resin. Electric energy is generated as a result of the tension or compression in this layer. The second layers should contain a resin with well-dispersed low concentration of fine piezoelectric particles. Electric energy is transferred from the first layer to the electrode on the second layer through conductive paths within the second layer
- A sedimentation process can be used for manufacturing of the particulate composite, which contains the above-mentioned two layers. Because of bi-modal powder size distribution and agglomeration effect, larger PZT particles and agglomerates quickly sink to the bottom of the liquid resin (bottom layer). Due to the viscosity and rate of cross-linking of the resin, very fine particles remain in the upper layer of the produced composite, creating randomly distributed conductive paths (top layer)
- A change in bottom layer thickness in the investigated composite results in change of the neutral axis position. Thickness of the whole manufactured composite should be selected so as to locate the neutral axis between the top and bottom layers.

## Figures and Tables

**Table 1:** Material parameters of the composite layers.

Parameter	PZT Grains Layer	Polymer Layer
Young modulus, E [GPa]	2.81	1.72
Poisson ratio, ν [−]	0.3	0.36
Dielectric constant, $\varepsilon_{r}\;\left[-\right]$	28.5	3
